# Milk is not just food but most likely a genetic transfection system activating mTORC1 signaling for postnatal growth

**DOI:** 10.1186/1475-2891-12-103

**Published:** 2013-07-25

**Authors:** Bodo C Melnik, Swen Malte John, Gerd Schmitz

**Affiliations:** 1 Department of Dermatology, Environmental Medicine and Health Theory, University of Osnabrück, Sedanstrasse 115, D-49090, Osnabrück, Germany; 2 Institute of Clinical Chemistry and Laboratory Medicine, University Clinics of Regensburg, Josef-Strauss-Allee 11, D-93053, Regensburg, Germany

**Keywords:** Branched-chain amino acids, Diseases of civilization, Glucose-dependent insulinotropic polypeptide, Glucagon-like peptide-1, Exosomal microRNA, Leucine, MicroRNA-21, Milk, mTORC1, Postnatal growth, Tryptophan

## Abstract

Milk has been recognized to represent a functionally active nutrient system promoting neonatal growth of mammals. Cell growth is regulated by the nutrient-sensitive kinase *mechanistic target of rapamycin complex 1* (mTORC1). There is still a lack of information on the mechanisms of mTORC1 up-regulation by milk consumption. This review presents milk as a materno-neonatal relay system functioning by transfer of preferential amino acids, which increase plasma levels of glucose-dependent insulinotropic polypeptide (GIP), glucagon-like peptide-1 (GLP-1), insulin, growth hormone (GH) and insulin-like growth factor-1 (IGF-1) for mTORC1 activation. Importantly, milk exosomes, which regularly contain microRNA-21, most likely represent a genetic transfection system enhancing mTORC1-driven metabolic processes. Whereas human breast milk is the ideal food for infants allowing appropriate postnatal growth and species-specific metabolic programming, persistent high milk signaling during adolescence and adulthood by continued cow´s milk consumption may promote mTORC1-driven diseases of civilization.

## Introduction

Milk is a highly specialized, complex nutrient system developed by mammalian evolution to promote postnatal growth. In contrast to feeding artificial infant formula, only human milk allows appropriate metabolic programming and protects against diseases of civilization in later life [1]. However, continued consumption of cow´s milk and dairy products during adolescence and adulthood is an evolutionarily novel behavior that may have long-term adverse effects on human health [2]. It is the intention of this review article to unravel milk´s functionality as a signaling system of evolution. The mechanisms of milk signaling presented here have been elucidated by translational research of the endocrine effects of cow´s milk consumption as well as individual protein components of bovine milk (whey protein and casein) on human subjects. The crucial function of milk of all mammals is to promote postnatal growth and to assure appropriate species-specific postnatal metabolic programming. On the molecular level, cell growth, cell proliferation, protein- and lipid synthesis, anabolic metabolic processes, and inhibition of autophagy are mediated by the nutrient-sensitive kinase *mechanistic target of rapamycin complex 1* (mTORC1) [3-5]. mTORC1 is activated by branched-chain amino acids, especially leucine, the most abundant amino acid of whey proteins, growth factors like insulin and insulin-like growth factor-1 (IGF-1), and sufficient cellular energy sensed by AMP-activated kinase (AMPK) [3,5,6]. Cow´s milk (subsequently termed “milk”) appears to promote mTORC1 signaling by providing amino acids that function as endocrine messengers to increase IGF-1 and insulin secretion as well as by milk-derived exosomal regulatory microRNAs (miRs), especially miR-21, which attenuates the inhibitory effects of various tumor suppressor proteins like phosphatase and tensin homolog (PTEN), Sprouty 1 and 2 and programmed cell death 4 (PDCD4) on mTORC1-signaling.

### Amino acid signaling of milk

#### Tryptophan-GH-IGF-1-mTORC1 pathway

Milk provides substantial amounts of tryptophan easily hydrolyzed from α-lactalbumin in milk´s whey protein fraction. Tryptophan promotes pituitary serotonin synthesis [7], which increases growth hormone (GH) secretion [8]. GH stimulates hepatic IGF-1 synthesis. Both, GH and IGF-1 have been shown to increase by milk consumption [9]. Casein proteins are rich sources of tryptophan, too. Casein in comparison to whey protein has been shown to differentially increase hepatic IGF-1 synthesis [10]. There is substantial epidemiological evidence that milk consumption efficiently elevates IGF-1 plasma levels by 20 to 30% in comparison to non-dairy consumers [9-14].

#### Leucine-insulin-mTORC1 pathway

Water soluble, easily hydrolysable whey proteins in comparison to all other animal-derived structural muscle proteins provide highest amounts of the branched-chain amino acids (BCAAs) leucine, isoleucine and valine, which raise postprandial insulin plasma levels within minutes [15-17]. Furthermore, whey proteins induce the secretion of the incretin *glucose*-*dependent insulinotropic polypeptide* (GIP), which in concert with insulinotropic BCAAs co-stimulates insulin secretion of pancreatic β-cells [15,16]. Milk proteins, especially leucine, stimulate the release of the intestinal incretin glucagon-like peptide-1 (GLP-1) [18]. It has previously been shown that leucine stimulates insulin secretion by β-cells due to its metabolism by oxidative decarboxylation and the ability of leucine to allosterically activate glutamate dehydrogenase (GDH) by β-cell mitochondria [19-21]. Xu et al. [22] demonstrated that leucine induced translation initiation by phosphorylation of 4E-BP-1 (formerly termed PHAS-I) and S6K, through the mTORC1-signaling pathway of pancreatic β-cells. In β-cells, leucine activates mTORC1 [19,20] that regulates insulin secretion and β-cell mass expansion [23-25]. Leucine not only increases insulin secretion but also enhances insulin signaling in insulin target tissues [26]. Chronic leucine supplementation elevated basal IRS-1 phosphorylation on tyrosine 632 and improved insulin-stimulated Akt and mTOR phosphorylation in liver, skeletal muscle and adipose tissue of rats fed a high fat diet [26]. In human skeletal muscle direct evidence has been provided that whey protein intake raised mTORC1 activity [27]. Thus, milk-derived BCAAs, especially leucine, appear to function as important messengers of mammalian lactation promoting insulin secretion and β-cell mass expansion required for appropriate mTORC1-driven postnatal growth.

#### Tryptophan-GIP-GH-IGF-1-mTORC1 pathway

**Figure 1:**
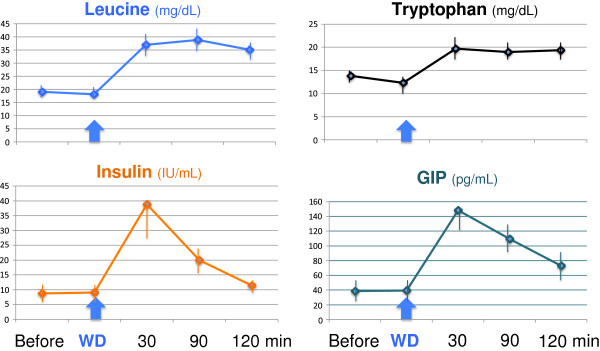
Mean (± SD) postprandial leucine, tryptophan and insulin serum levels and postprandial GIP plasma levels after a 30 g whey protein drink (WD) of 10 healthy volunteers (8 males, 2 females, mean age 25.6 ± 6.0 yrs).

Tryptophan deficiency has profound inhibitory effects on protein synthesis, RNA translation and growth [28]. Intragastric addition of tryptophan to early-weaned piglets increased intestinal GIP secretion [29]. Whey proteins and caseins are rich protein sources of tryptophan. Test meals of 16.7 g and 18.2 g whey protein to healthy young adults substantially increased GIP secretion and postprandial plasma GIP concentrations [15,16], further supported by own data on postprandial plasma GIP levels of 10 healthy young adults (8 males, 2 females, mean age 25 yrs) after 30 g whey protein intake (Figure 1). Hydrolyzed peptides of whey protein competitively inhibit the GIP inactivating enzyme dipeptidyl peptidase IV, thereby extending GIP bioactivity [30]. GIP may not only signal via the entero-insular axis stimulating insulin secretion but also enhances GH secretion of the somatotroph cells of the pituitary, which express the GIP-receptor (GIPR) [31]. GIPR activation elevates cAMP, which drives GH-promoter activity [31]. Thus, GIP not only responds to dietary glucose but may function as a whey (tryptophan)-dependent GH-stimulating hormone that activates both pancreatic insulin as well as hepatic IGF-1 synthesis for mTORC1-dependent protein and lipid synthesis required for cell growth. Remarkably, deletion of tryptophan from a hepatocyte culture medium substantially decreased IGF-1 synthesis [32]. In accordance, Rich-Edwards *et al*. [9] demonstrated that milk consumption of children increased serum GH and IGF-1 levels and shifted the somatotropic axis to higher levels. Furthermore, it has been demonstrated in ovine hepatocyte cultures that IGF-1 synthesis clearly depends on amino acid availability in a dose dependent manner [33]. In a rat hepatocyte primary culture, IGF-1 mRNA expression was dependent on amino acid availability [34]. Furthermore, the essential amino acid content of the diet is critical for the optimal restoration of IGF-1 after fasting, when protein intake is reduced [35]. Recent evidence has been provided that post-exercise replenishment of essential amino acids plus carbohydrate significantly increased leucine and free IGF-1 serum levels of 8 young healthy males [36]. Insulin increases hepatic IGF-1 synthesis and enhances free IGF-1 bioactivity by inhibition of hepatic insulin-like growth factor-binding protein-1 (IGFBP-1) [37-39]. IGFBP-2 may be subject to dual control, with GH and amino acid availability serving as the primary regulators [40,41]. Thus, milk-derived amino acids provide a sophisticated regulatory network (GH, insulin and amino acids, especially leucine and tryptophan) that stimulates downstream IGF-1-signaling.

#### Amino acid-IGF-1-insulin-mTORC1 pathway promoting cell growth

As demonstrated above milk-derived amino acids up-regulate the secretory activity of the pituitary gland (GH secretion), the liver (IGF-1 secretion), the pancreatic β-cells (insulin secretion), the intestinal enteroendocrine K-cells (GIP secretion), and L-cells (GLP-1 secretion). Milk proteins in comparison to all other animal proteins provide the highest postprandial levels of the BCAAs leucine, isoleucine and valine [15,16,42]. Milk-driven insulin/IGF-1 signaling combined with leucine abundance provides optimal conditions for up-regulation of mTORC1 mediating accelerated growth and proliferation of peripheral cells of the milk recipient. Persistent milk consumption and dairy-enriched Western diet thus represents a fundamental stimulus for continued mTORC1 activation with all its adverse consequences in adolescence and adulthood [43].

### MicroRNA signaling of milk

#### The role of milk´s exosomal microRNAs

Secreted microRNAs (miRs) represent a newly recognized most important layer of gene regulation in eukaryocytes, which plays a relevant role for intercellular communication [44,45]. miRs bind through partial sequence homology to the 3′-untranslated region of target mRNAs and cause either translational block or less frequently mRNA degradation [46]. miRs that are enclosed by membranous microvesicles, so-called exosomes, play a pivotal role for horizontal miR transfer [47]. Intriguingly, breast milk contains the highest concentration of total RNAs (47,240 μg/L) in comparison to other body fluids like plasma (308 μg/L) [48]. There is accumulating evidence that bovine and human milk transfer substantial amounts of miRs for regulatory functions by exosomal transport [49-51]. Chen *et al*. [50] have detected 245 miRs in cow’s milk. They reported relatively high and consistent expression of seven miRs in mature cow´s milk at various lactation periods listed from highest to lowest sequencing frequencies: miR-21 (24,137), miR-99a (24,097), miR-148a (16,597), miR-30d (14,089), miR-200c (11,010), miR-26b (6,595) and miR-26a (3,376), respectively [50]. In cow´s milk, whey and bovine colostrum, miR-containing exosomes of 50–100 nm have been identified [52-54].

Milk exosomal miRs are of functional importance for the development and maturation of the neonate´s immune system [54-56]. These findings implicate that exosomal miRs of milk may reach the systemic circulation and organ systems. The exosome lipid membrane protects milk-derived miRs against degradation. Remarkably, miRs of cow´s milk are resistant against acidic conditions (pH2) as well as heat exposure and external RNAse treatment [51], most likely withstanding the harsh degrading conditions in the gastrointestinal tract. Although, raw milk contains the highest amounts of total miRs, pasteurized commercial milk and milk powder still contain substantial and stable concentrations of miRs [50]. It has already been suggested that, milk-derived exosomes may pass the intestinal barrier and reach the systemic circulation [51]. Intestinal cells release exosomes of 30–90 nm in diameter from their apical and basolateral sides [57]. The tetraspanin CD63, a known exosome marker of cow´s milk exosomes [52], is present on intestinal epithelial-derived exosomes [57]. CD63- and CD81-containing tetraspanin molecules have been detected on exosomes in human plasma [58]. In fact, blood is regarded as a physiological fluid for exosome circulation in the body, pointing to the important role of exosomes as carriers for cell-cell or organ-organ communications [58-60]. Furthermore, bovine milk´s predominant miR species, miR-21, is a major component of human plasma [61]. Thus, it is conceivable that milk´s exosomal miRs reach the plasma compartment to function as a messenger system promoting postnatal growth. In fact, it has already been demonstrated that a diet-derived miR, the plant MIR168a, reaches the plasma compartment of human subjects and affects LDLRAP1 metabolism in the liver [62]. Thus, there is good reason to assume that cow´s milk-derived miRs affect distant regulatory networks and organs of the milk recipient. A systemic transfer of milk-derived miRs to the neonate or the persistent milk consumer may augment mTORC1-mediated growth signaling, which is a physiologically required process for postnatal growth and development but not for humans after the lactation period.

#### Potential role of milk miR-21 for the augmentation of mTORC1 signaling

**Figure 2:**
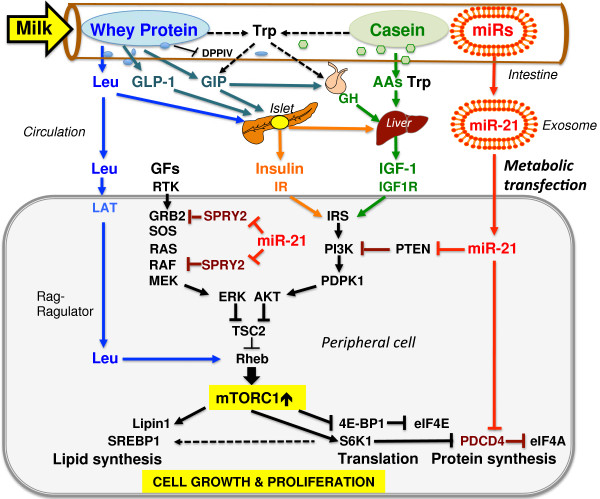
**Synoptic model of amino acid- and exosomal miR-mediated signaling of milk for the activation of mTORC1-mediated postnatal growth.** Whey protein (WP)-derived leucine, isoleucine and valine stimulate insulin synthesis. WP (especially Trp) induces the incretrin GIP, which will further enhance insulin synthesis. Peptide fragments of WP hydrolysis competitively inhibit DPPIV, thereby extending GIP bioactivity. GIP via GIP-R on somatotroph pituitary cells stimulates the synthesis of GH, which up-regulates hepatic IGF-1-synthesis, further augmented by insulin and Trp. Leucine derived from milk proteins increases GLP-1. Increased insulin/IGF-1 signaling via inhibition of TSC2 activates mTORC1. WP-derived leucine via Rag/Ragulator interaction promotes the activation of mTORC1. mTORC1 stimulates lipid synthesis by phosphorylation of lipin1 and activation of S6K1, which enhance lipogenesis. Milk is hypothesized to operate as an exosome-driven miR transfection system of metabolism to increase mTORC1-driven anabolic reactions of the milk recipient. Especially, milk exosomes containing miR-21 may enhance mTORC1 signaling by suppression of tumor suppressor proteins PTEN, Sprouty and PDCD4 (see list of abbreviations).

Exosomal miR-21, a consistent component of cow´s milk and human breast milk [50,56], appears to play a key role in mTORC1 signaling. Critical targets of miR-21 are mRNAs of important tumor suppressor proteins involved in upstream and downstream suppression of mTORC1 signaling, i.e., PTEN [63-66], Sprouty1 and Sprouty2 [67-69], PDCD4 [70-72] (Figure 2). Furthermore, miR-21 has been shown to induce the cell cycle promoter cyclin D1 in an mTORC1-dependent manner [73]. Supposed that milk-derived miR-21 reaches distant cells of the milk recipient, PTEN suppression could increase insulin/IGF-1/PI3K/Akt signaling, which further augments mTORC1 activation. miR-21-induced inhibition of Sprouty1 and 2 would amplify Ras-Raf-MEK-ERK signaling, which additionally suppresses TSC2 and thus raises mTORC1 activity (Figure 2). Furthermore, miR-21 could stimulate the initiation of translation by repression of PDCD4, which is a suppressor of translation initiation that inhibits the RNA helicase eIF4A [74]. Both, 4E-BP-1 and PDCD4 are crucial regulatory inhibitors of translation initiation and thus of protein synthesis. Activation of the mTORC1 pathway and its substrate kinase S6K1 results in subsequent phosphorylation of 4E-BP-1 and PDCD4 that promote eIF4E-eIF4G complex assembly and stimulate mRNA translation [74]. miR-21-mediated suppression of PDCD4 expression may further amply translation initiation, a reasonable regulatory step of milk signaling to promote postnatal growth. In this regard, miR-21 signaling of milk appears to enhance upstream and downstream mTORC1 signaling.

Thus, milk appears to combine both amino acid- and miR-mediated pathways to optimize mTORC1 signaling for the promotion of postnatal growth. However, it is of critical concern that miR-21 is a well-known oncogene, which by suppressing various tumor suppressor genes plays a key role in resisting programmed cell death [75]. In comparison to amino acid signaling of milk, which primarily affects layers of posttranslational modifications, miR signaling of milk may represent an even more powerful archaic regulatory network, because it interferes with posttranscriptional regulation of numerous genes and gene networks. In fact, miR-21 has been shown to contribute to renal cancer cell proliferation and migration via activation of mTORC1 [76].

#### Milk signaling and the promotion of diseases of civilization

There is accumulating evidence that chronic diseases of civilization are associated with increased mTORC1 signaling [77,78], like acne [79,80], obesity [81,82], type 2-diabetes [77,83], arterial hypertension [84], Alzheimer´s disease [85], cancer [86], especially prostate cancer [87,88]. Thus, cow´s milk is not just a simple food for humans, but a tremendously powerful evolutionary program of the faster growing species *Bos taurus*, which may permanently over-stimulate mTORC1 signaling in human milk consumers. In this regard the *milk kinship hypothesis* appears in a new light, which explains the increased risk for genetic disease in offspring of marriages of “milk siblings”, who were breastfed by the same woman (forbidden by the Qur´an in countries of the Middle East) [89]. Thus, we are at the beginning to ask for the metabolic consequences of bovine milk-derived miRs on human subjects during various phases of human growth and development.

#### Milk-mediated mTORC1-activation, adipogenesis and insulin resistance

Further research should investigate the precise trafficking of milk exosomes, which most likely reach the systemic circulation of the milk recipient. Continued mTORC1-activation by milk-derived exosomal transfer of miR-21 may represent a persistently adverse health effect of regular milk and dairy product intake, which may play an important role for the development and progression of mTORC1-driven diseases of civilization [77-88]. In analogy to the postulated *Trojan exosome hypothesis* explaining the role of exosomes for the spread of RNA viruses [90], the milk exosome system too appears to function as a Trojan horse “transfecting” the neonate´s metabolism to ensure species-specific mTORC1-driven growth and anabolism.

There is accumulating evidence that milk consumption in children, adolescents and adults increases body mass index (BMI) [81-83,91-93] and induces insulin resistance in children [17]. In Nordic countries, where women regularly consume milk during pregnancy, increased infant birthweights related to dairy protein intake during pregnancy have been reported [94,95].

In comparison to all other structural animal proteins, whey proteins provide highest amounts of most easily hydrolysed BCAAs [42], which raise postprandial plasma BCAA levels within minutes [15,16]. Thus, other high BCAA sources like muscle proteins of beef differ from soluble milk proteins, especially whey, by a more retarded release of BCAAs into the systemic circulation. In fact, beef has an insulinemic index of 51 [96], whereas insulin scores for milk and milk products range from 89 to 115 [96,97]. Increased BCAA plasma levels in children and adolescents have recently been associated with increased risk for elevated BMI, insulin resistance and type 2 diabetes mellitus [98,99]. However, the most striking difference between milk and muscle or plant proteins is the high amount of specific exosomal miRs, like miR-21 and miR-103 [50,51], that may play an important role as an additional layer of metabolic regulation.

### Potential role of milk-derived miRs in metabolic regulation

There is accumulating evidence that miRs are highly connected nodes in regulatory networks underlying adipogenesis and adipose dysfunction in obesity [100]. Kim et al. [101] demonstrated that miR-21 regulates adipogenic differentiation in mesenchymal stem cells derived from human adipose tissue. The same group observed a correlation between miR-21 levels and adipocyte number in white adipose tissue (WAT) of high fat-diet induced obese mice [102]. The later study suggests that miR-21 may control the proliferation of adipocyte precursors [102]. There is further compelling evidence that adipogenesis depends on mTORC1 signaling [103]. As miR-21 reduces the expression of various tumor suppressor genes of the mTORC1 signaling pathway, milk-derived exosomal miR-21 may promote adipogenesis. In fact, genetic deletion of S6K1 in mice (S6K1^−/−^ mice), the downstream target of mTORC1, inhibited the transformation of mesenchymal stem cells into adipocytes and reduced the total number and size of adipocytes [104]. Ectopic expression of miR-103 in preadipocytes accelerated adipogenesis as measured by both the up-regulation of many adipogenesis markers and by an increase in triglyceride accumulation at an early stage of adipogenesis [105]. miR-103 is a component of cow´s milk [51] (Table 1).

**Table 1:** Selected milk-derived miRs and their potential impact on metabolism

**Milk-derived miR**	**Ref.**	**miR function in metabolism**	**Ref.**
miR-21	50	miR-21: Inhibition of various tumor suppressor gene mRNAs (PTEN, Spouty1, Sprouty2, PDCD4)	[63-72,75]
		Increased adipogenesis	[100-102]
miR-29a	54	miR-29a: Down-regulation of Insig-1 with increased lipogenesis and adipocyte differentiation	[106]
			[107,108]
miR-29b	51	miR-29b: Reduction of BCKD and reduced BCAA catabolism	[111]
			[110]
miR-103	51	miR-103: Increased adipogenesis	[100,105]
miR-155	51	miR-155: Reduction of brown adipose tissue and thermogenesis	[109]
Let-7a, b, c, f	51	Let-7: overexpression results in insulin resistance and disturbed glucose homeostasis	[120,122]

Insulin-induced gene 1 (insig-1) mRNA is a validated target of miR-29a [106], a further miR detected in cow´s milk [54]. Insig-1 binds sterol regulatory element-binding protein (SREBP) cleavage-activating protein in the endoplasmic reticulum, thereby blocking proteolytic processing required for SREBP activation [107]. Insig-1 restricts lipogenesis in mature adipocytes and inhibits differentiation in preadipocytes [108]. Inactivation of insig-1 mRNA by milk-derived miR-29a may thus be another potential mechanisms by which milk consumption my promote adipogensis and BMI increase as observed in children, adolescents and adults [81-83,91-93].

Remarkably, cow´s milk contains substantial amounts of miR-155 [51]. The target of miR-155 is the adipogenic transcription factor *CCAAT*/*enhancer*-*binding protein β* (C/EBPβ). Overexpression of miR-155 in mice has been shown to reduce brown adipose tissue (BAT) mass [109]. Thus, milk miR-155 intake may attenuate thermogenesis of BAT, an unfavorable condition promoting lipid and energy storage in WAT promoting obesity. However, attenuation of BAT thermogenesis may be a developmentally appropriate postnatal event.

Elevated plasma BCAAs have been associated with reduced cellular BCAA utilization and/or incomplete BCAA oxidation [110]. The mitochondrial BCAA oxidation checkpoint, which commits BCAAs to degradation is the branched-chain α-ketoacid dehydrogenase (BCKD) complex. BCKD activity is reduced in WAT of obese individuals [110]. miR-29b is targeted to the mRNA for the dihydrolipoamide branched chain acyltransferase component of BCKD and prevents translation when bound [111]. Thus, miR-29b inhibits the pathway of BCAA catabolism. Intriguingly, miR-29b is a major miR of bovine milk [51]. Milk´s miR-29b may thus function to maintain high plasma BCAAs levels important for mTORC1-dependent growth and appropriate amino acid uptake for protein de novo biosynthesis of functional and structural proteins required for postnatal growth. High plasma levels of milk-derived BCAAs together with milk-miR-29b-mediated inhibition of BCAA catabolism may thus over-activate mTORC1-S6K1 signaling. Insulin resistance during mTORC1-driven phases of growth may represent a negative feedback mechanism, induced by up-regulated S6K1, which via IRS-1 phosphorylation inhibits downstream insulin signaling [112]. These metabolic events may explain the link between elevated BCAA plasma levels and insulin resistance [113]. In fact, Hoppe *et al*. demonstrated that milk consumption but not meat (which misses miR-29b) induced insulin resistance in Danish prepubertal boys [17]. Thus, milk, the starter kit of mammalian evolution, appears to execute a highly sophisticated metabolic program, which orchestrates BCAA-, miR-21- and miR-29b-driven mTORC1-mediated protein biosynthesis. In synergy with activated protein biosynthesis, the mTORC1-driver milk should promote lipid synthesis too. Remarkably, it has recently been recognized that activated mTORC1 induces the expression of key transcription factors of lipogenesis, sterol response element binding protein-1 (SREBP-1) [4,114-116], and peroxisome proliferator-activated receptor-γ (PPARγ) [117,118]. Furthermore, activated mTORC1 has been shown to suppress lipolysis, stimulates lipogenesis and promotes fat storage [119]. Thus, milk appears to provide an endocrine signaling environment for increased mTORC1-driven lipogenesis and fat storage as well as miR-155-induced suppression of BAT differentiation resulting in BMI elevations and fat deposition in WAT [81-83,91-93].

Milk contains substantial amounts of let7a, let7b, let7c and let7f [50]. There is accumulating evidence that the Lin28/let-7 axis regulates glucose metabolism [120]. Muscle specific loss of Lin28a and overexpression of let-7 resulted in insulin resistance and impaired glucose tolerance in mice [120]. Intriguingly, let-7 targets are enriched for genes that contain SNPs associated with type 2 diabetes and fasting glucose in human genome-wide association studies [120]. Lin28, a developmentally regulated RNA-binding protein, selectively blocks the processing of pri-let-7 miRs [121]. Notably, the restoration of the Lin28 protein blocked let-7 expression and restored glucose metabolism in adipose-derived stem cells derived from obese tissues [122]. The most interesting miRs in inflammatory microvesicles in association with metabolic and cardiovascular diseases recently reported are the let-7 family, miR17/92 family, miR-21, miR-29, miR-126, miR-133, miR-146 and miR-155 [123]. Notably, there is a substantial overlap with miRs derived from milk exosomes (Table 1).

## Conclusions and future perspectives

Routine milk consumption, which has been boosted by the introduction of refrigeration technology in the early 1950´s, is an evolutionarily novel dietary behavior of *Homo sapiens* of the Neolithic period, which may have adverse long-term biological consequences [2]. Milk is not just food but appears to represent a most sophisticated endocrine signaling system activating mTORC1 via special maternal milk-derived dietary messengers controlled by the mammalian lactation genome: BCAAs of milk proteins and exosomal miRs produced by the mammary gland, which appear to augment mTORC1 signaling for postnatal growth. In this regard, it is of critical concern that persistently increased mTORC1 signaling has been recognized as the fundamental driving force for the development of mTORC1-driven diseases of civilization [77-83]. Therefore, future research in nutrition science should pay special attention to the function of milk-derived BCAAs and furthermore should clarify the potential role of milk´s exosomal miR-transfer on metabolic regulation in the milk recipient. The potential uptake of labeled exosomal miRs derived from commercial milk has to be studied in animal models and humans in greater detail.

## Abbreviations

AAs: Amino acids; AMP: Adenosine monophosphate; AMPK: AMP-activated protein kinase; Akt: V-AKT murine thymoma viral oncogene homolog (protein kinase B); BAT: Brown adipose tissue; BCAA: Branched-chain amino acid; BCKD: Branched-chain α-ketoacid dehydrogenase; BMI: Body mass index; C/EBPβ: CCAAT/enhancer-binding protein β; DPPIV: Dipeptidyl peptidase IV; 4E-BP-1: Eukaryotic translation initiation factor 4E-binding protein 1; eIF4A: Eukaryotic translation initiation factor 4A; eIF4E: Eukaryotic translation initiation factor 4E; ERK: Mitogen-activated protein kinase kinase 4; GDH: Glutamate dehydrogenase; GF: Growth factors; GH: Growth hormone; GHR: Growth hormone receptor; GIP: Glucose-dependent insulinotropic polypeptide; GIPR: GIP receptor; GLP-1: Glucagon-like peptide-1; GRB2: Growth factor receptor-bound protein 2; IGF-1: Insulin-like growth factor-1; IGF1R: Insulin-like growth factor-1 receptor; IR: Insulin receptor; IRS: Insulin receptor substrate; PI3K: Phosphoinositide-3 kinase; LAT: L-type amino acid transporter; Leu: Leucine; miR: micro-ribonucleic acid; MEK: Mitogen-activated protein kinase kinase 1; mTORC1: Mechanistic (mammalian) target of rapamycin complex 1; PDCD4: Programmed cell death 4; PDPK1: 3-Phosphoinositide-dependent protein kinase 1; PTEN: Phosphatase and tensin homolog; RTK: Receptor tyrosine kinase; Raf: V-RAF-1 murine leukemia viral oncogene homolog; Ras: V-HA-RAS rat sarcoma viral oncogene homolog; Rheb: Ras homolog enrich in brain; S6K1: Ribosomal protein S6 kinase, 70-kD kinase 1; SOS: Son of sevenless; SPRY2: Sprouty2; Trp: Tryptophan; SREBP-1: Sterol regulatory element-binding transcription factor 1; TSC2: Tuberin; WAT: White adipose tissue.

## Competing interests

The authors declare that they have no competing interests.

## Authors’ contributions

BCM wrote the manuscript and formulated the hypothesis of milk exosomal miR-mediated transfection of metabolism of the milk recipient. GS evaluated postprandial plasma parameters after whey protein challenge of human volunteers, researched data and contributed to the discussion and conclusions. BCM and SMJ both performed the whey protein study with 10 healthy volunteers. All authors read and approved the final manuscript.
